# Factors associated with uterine fibroid in Ghanaian women undergoing pelvic scans with suspected uterine fibroid

**DOI:** 10.1186/s40738-016-0022-9

**Published:** 2016-05-01

**Authors:** Benjamin Dabo Sarkodie, Benard Ohene Botwe, David Nana Adjei, Eric Ofori

**Affiliations:** 1 Univeristy of Ghana, School of Medicine and Dentistry, P. O. Box GP 4236, Accra, Ghana; 2 Department of Radiography, University Ghana School of Biomedical and Allied Health Sciences, College of Health Sciences, P.O. Box KB 143, Korle Bu, Accra, Ghana; 3 Department of Medical Laboratory Sciences, University Ghana School of Biomedical and Allied Health Sciences, College of Health Sciences, P.O. Box KB 143, Korle Bu, Accra, Ghana

**Keywords:** Uterine fibroid, Ultrasound, Ghana, Associated factors

## Abstract

**Background:**

Uterine fibroids are the most common benign tumours affecting premenopausal women and are often associated with considerable hospitalization and morbidity. Literature shows virtually no study concerning the quantification of the main factors associated with uterine fibroids in Ghanaian women. The purpose of this study was to assess the main factors associated with uterine fibroid among Ghanaian women presenting for ultrasound.

**Method:**

A prospective cross-sectional study design was employed in this study. A total of two hundred and forty-four (244) women were consecutively evaluated from November 2011 to February 2012 using a 2–5 MHz curvilinear probe of Philips HD3 ultrasound machines at three centres in Accra using a trans-abdominal pelvic approach. Data was analysed with (SPSS) version 20.0 for windows, 2010; Chicago. The Pearson’s Chi-square test was used to determine associations between selected demographic and gynaecological characteristics and uterine fibroid appearance. All tests were two-tailed and p-value of less than 0.05 was interpreted as significant.

**Results:**

The range, mean and standard deviation (SD) of the patients’ age were 14–54 years, 31.89 years and ± 7.92 respectively. Factors that associated significantly with uterine fibroid in Ghanaian women included obesity (*X* ^2^ = 17.3, *p*-value = 0.001), participant’s age range (*X* ^2^ = 47.4, *p*-value = 0.001), parity (*X* ^2^ = −10.169, *p*-value = 0.001), and age at last delivery (*X* ^2^ = 34.579, *p*-value = 0.001).

**Conclusion:**

Uterine fibroid was mainly associated with women of older age group of the reproductive age than the younger age categories and also associated more with women with without children compared to those with more children. Moreover, it associated more with obese patients and patient with late age at last delivery.

## Background

Uterine fibroids, also known as uterine leiomyomata, are mostly benign growths that develop in the muscles of the uterine wall [1]. They are non-cancerous tumours which are myometrial in origin [2]. It is uncommon for fibroids to develop into cancer (leiosarcoma) and occurs in < 0.1 % of cases [3]. Fibroids grow from tiny uterine muscle cells and may be initially diagnosed by an imaging procedure as small nodules and can grow to larger sizes where they are palpated through the abdominal wall with variable sizes even beyond 7.5 cm [4]. Fibroid can be considered as well-defined masses emanating from the smooth muscle layer of the uterus and also composed of extracellular matrix and considered to be the most common tumour in the female pelvis [5]. Uterine leiomyoma, represent a major public health problem and the most common benign gynaecologic tumours affecting premenopausal women [6] and they are often associated with considerable morbidity [7]. Uterine fibroids have also been associated with spontaneous miscarriages [8]. They are the most frequent indication for hysterectomy (abdominal and vaginal), accounting for approximately one third of all procedures performed annually in the United States. It is suggested that black women have a greater fibroid burden than whites [9–12].

Uterine leiomyoma, or fibroids, are the most common tumours of women in the United States, probably occurring in the majority of women by the time they reach menopause and becoming clinically significant in about one third of these women [7]. Despite their prevalence, little attention has been directed toward the aetiology and pathogenesis of fibroids until recent years because of the rarity of their malignant transformation [7]. Regardless of their generally benign neoplastic character, uterine fibroids are responsible for significant morbidity in a large segment of the female population. The clinical effects of these tumours are related to their local mass effect, resulting in pressure upon adjacent organs, excessive uterine bleeding, or problems related to pregnancy, including infertility and repetitive pregnancy loss [13]. As a consequence of these local pressure effects and bleeding, uterine fibroids rank as the major reason for hysterectomy in the United States, accounting for approximately one-third of all hysterectomies [9] or about 200,000 hysterectomies per year. Although the aetiology of fibroid is unknown, the scientific literature now contains a sizeable body of information pertaining to the epidemiology, genetics, hormonal aspects, and molecular biology of these tumours.

According to Goodwin & Spies [14], the ovarian hormones oestrogen and progesterone are hypothesized to enhance fibroid growth [14]. Reported risk factors consistent with the hormonal hypothesis include premenopausal status [15, 16] younger age at menarche [15, 17] and obesity [15, 18]. Histories of infertility, young age at first birth, and current alcohol consumption have been associated with increased risk [15]. Reported protective factors include parity [15, 17] and oral contraceptive use [19, 20]. Use of oral contraceptives at young ages was reported to be associated with an increased risk in at least one study [17]. Cigarette smoking has been reported to be inversely related to risk in several studies [15, 21, 22], but not all [23]. These findings stemmed largely from case control studies among which eligibility criteria (i.e., age, race, marital status) varied, as did case definitions (i.e., self-reports; histologic, pathologic, or ultrasound/hysterectomy confirmation).

Although the initiator or initiators of fibroids are unknown, several predisposing factors have been identified, including age (late reproductive years), African-American ethnicity, nulliparity, and obesity [11]. A population-based study in the United States found a cumulative incidence of uterine fibroids of greater than 66 % by ultrasound examination of women approaching age 50 years [24].

A study by Faerstein [25] showed that parity reduces risk of fibroid by 20–40 % and the risk further reduces as parity increases. However, Parrizziani [26] indicated that induced abortion increases the risk of developing fibroid nodules. This notwithstanding, a study by Segars [27] suggested that trauma or a sort of trigger in the uterus causes uterine cells to contract abnormally and that could initiate the formation of fibroid nodules. People with hypertension, atherosclerosis and heart related disease have shown increased risk of fibroid and by extension it was noted that high blood pressure was associated with high risk of clinical diagnosis of fibroid even after giving the appropriate medical care and treatment for high blood pressure [28]. According to Wise et al. [29], increase in age was noted as an outstanding risk factor for uterine fibroid. In another study, smoking is noted to cause reduction in the levels of oestrogen and body weight which is associated with low incidence of fibroids [26]. Furthermore, Baird et al. [30], revealed that physical activity reduces the risk of developing fibroid by 40 %. Apart from the risk factors listed above, there are other factors which have not been extensively investigated which include environmental and dietary factors [4].

Literature search both electronic and manual indicated a gap in terms of documentation on the main factors associated with uterine fibroids in Ghanaian women. In Ghana, this gab in information appears to limit some effort to promote and create awareness on fibroid among the populace. According to the Ghana Statistical Service, the female population in Ghana is higher than the male population, which indicates that the female population in the work force will also increase. Premenopausal women (18–45 years) in Ghana constitute about 40 % of the population [31]. Therefore, more women will be absent from work due to fibroid related problems if it is not looked into, which will eventually affect productivity and the economy as a whole. It is therefore imperative to ensure continuous availability of documented data on the subject that may serve as useful source of information for policy development and management of uterine fibroids. It is against this backdrop that this study was undertaken to assess the associated factors of uterine fibroid among Ghanaian women presenting for ultrasound scans.

## Method

A cross-sectional convenience study, evaluating trans-abdominal pelvic ultrasound reports generated prospectively in three diagnostic centres in the Accra metropolis, Ghana was the study design. The centres were chosen because they had the same equipment models, and large patient turn over, from across the country. The study was conducted between November 2011–February 2012. Two hundred and forty four women in their reproductive age who were concurrently referred to the centres for pelvic ultrasound examination on account of suspected fibroids were recruited for the study. Thus, women who had attained menopause as well as those yet to attain menarche were excluded.

### Data analysis

The data collected was collated and analysed using the Statistical Package for Social Sciences (SPSS) version 20.0 for windows, 2009; Chicago. Descriptive statistics were calculated. The Pearson’s Chi-square test was used to determine associations between selected demographic and gynaecological characteristics and fibroid appearance. All tests were two-tailed and *p*-value of less than 0.05 was interpreted as significant.

### Ethical considerations

In order to ensure that the data from the various interest groups obtained were handled in an ethical manner, the following measures were taken. Ethical clearance was sought and obtained from the Ethics committee of Ghana Medical School, College of Health Sciences, University of Ghana. Approval was also sought from the Medical Directorate of the selected centres.

Because cooperation and commitment of the patients’ involved in the project is crucial, discussions were held with them prior to the study. This decision was taken in order to get (i) their individual consent and (ii) to encourage them to participate voluntarily and encourage co-operation.

Since additional data (gynaecological characteristics) other than that provided on patients’ request forms was recorded, patients were made aware of the importance of the study for which data was being taken and the need for their cooperation and consent. They were also informed that the data being taken would not in any way, affect the results of their examination to be done. Additionally, they were assured of confidentiality of their identity and the information collected on them. Finally, patients signed a consent form signifying that they had agreed to the use of the data collected for research purposes. Those who could not sign gave verbal consent. 

### Machine/technique and protocol

The ultrasound scanning machines used at the three study sites were the same. Table 1 shows the characteristics of Ultrasound scanners available in the centres.

**Table 1:** Parameters of the equipment used

Parameter of the equipment	Details
Type	HD
Manufacturer	PHILIPS MEDICAL
Model	HD3
Year of manufacture	2005
Probe	Curvilinear
Frequency of probe	2–5 MHz

Technical specifications of the ultrasound scanning equipment used in all three (3) centres are shown in Table 1.

## Results

**Fig. 1:**
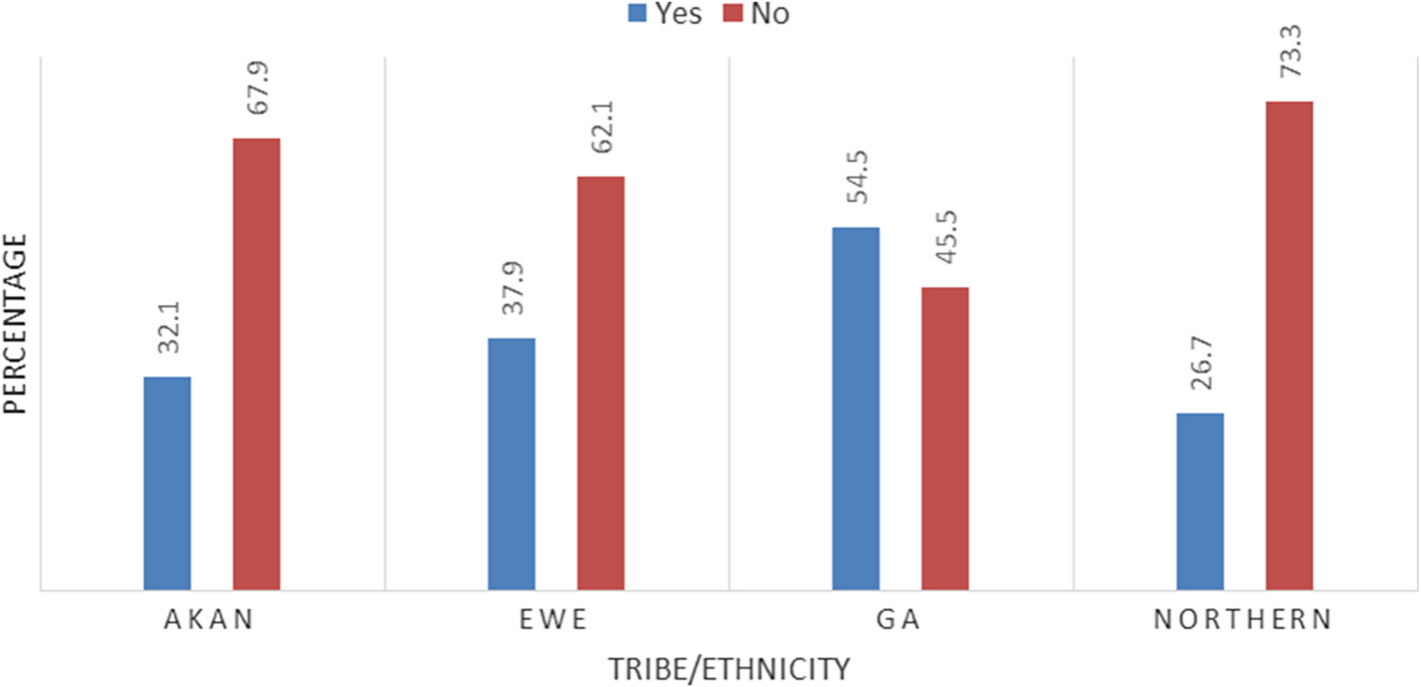
Confirmed fibroid distribution among the ethnic groups

Two-hundred and forty-four (244) patients, who satisfied the inclusion criteria, were evaluated by this study. The age range, mean and standard deviation (SD) of patients were 14–54 years, 31.9 years and ± 7.9 respectively. Close to half 112 (45.9 %) of the patients were Akans while only 30 (12.3 %) were from the Northern region. The patients’ demographics and gynaecological characteristics are presented in Table 2. From the study, 23 % (38/168) of women <35 had prevalent fibroids, compared to 67 % (36/54) of women 35–44, and 73 % (16/22) of women at 45 or above years. Among the ethnic groups, 54.5 % of Ga, 37.9 % of Ewes, 32.1 % of Akan and 26.7 of Northerners’ had uterine fibroids (Fig. 1).

**Table 2:** Characteristics of patients

Characteristics	Number	Percent
Age group (years)		
14–24	44	18.0
25–34	54	22.1
35–44	124	50.8
45 and above	22	9.0
Total	244	100
Highest educational level		
Primary	14	5.7
Junior High School	64	26.2
Senior High School	54	22.1
Tertiary	96	39.3
Post-Graduate	14	5.7
Uneducated	2	0.8
Total	244	100
Tribe		
Akan	112	45.9
Ewe	58	23.8
Ga	44	18.0
Northern	30	12.3
Total	244	100
Parity		
Zero	156	63.9
1 and above	88	36.1
Total	244	100
Body Mass Index		
<25	76	31.1
25–29.9	78	32.0
30 and above	90	36.9
Total	244	100
Menstrual flow pattern (heavy flow)		
Yes	102	41.8
No	142	58.2
Total	244	100
Type of menstrual cycle (irregular)		
Yes	164	67.2
No	80	32.8
Total	244	100

### Associations between selected demographics and presence of uterine fibroids

Table 3 shows associations between selected demographic, gynecological characteristics and the presence of uterine fibroids. There was significant positive association between age group (*X* ^2^ = 47.4, *p*-value = 0.001) and BMI (obesity) (*X* ^2^ = 17.3, *p*-value = 0.001) of patients and presence of uterine fibroids. Parity (*χ* ^2^ = −10.169, *p* = 0.001) showed a negative association with fibroids. Other factor that showed positive association with fibroid was age at last delivery (*X* ^2^ = 34.579, *p*-value = 0.001).

**Table 3:** Factors associated with uterine fibroids among participants diagnosed of having fibroids and their levels of significance

Characteristic		Confirmed diagnosis of fibroids in participants		χ^2^ value	*P*-value
		Yes (%)	No (%)		
Age Range	14–24	0 (0)	44 (28.6)	47.4	0.001
	25–34 years	38 (42.2)	130 (84.4)		
	35–44 years	36 (40.0)	18 (11.7)		
	45 years and above	16 (17.8)	6 (3.9)		
BMI	Normal	18 (20.0)	56 (36.8)	17.3	0.001
	Overweight	22 (24.4)	52 (34.2)		
	Obese	50 (55.6)	44 (28.9)		
Parity	Zero/Nulliparous	44 (48.9)	44 (28.6)	−10.169	0.001
	1 and above	46 (51.1)	110 (71.4)		
Age at 1^st^ Menstruation	Less than 12 years	2 (2.4)	8 (6.1)	11.191	0.0600
	12–15 years	44 (53.7)	94 (71.2)		
	16 years and above	36 (43.9)	30 (22.7)		
Age (Years) at Last Delivery of Participants	Nulliparous	42 (46.7)	100 (64.9)	14.603	0.004
	<24 years	6 (6.7)	18 (11.7)		
	25–29 years	14 (15.6)	14 (9.1)		
	30 years and above	28 (31.1)	22 (14.3)		
Type of Menstrual Cycle of Participants (Irregular Cycle)	Yes	62 (68.9)	102 (66.2)	0.182	0.670
	No	28 (31.1)	52 (33.8)		
Presence of severe pains during menstruation	Yes	62 (68.9)	82 (53.2)	0.471	0.493
	No	38 (42.2)	72 (46.8)		
Age at 1st Delivery	Less than 18 years	4 (8.3)	6 (11.1)	2.456	0.402
	18–34 years	42 (87.5)	48 (88.9)		
	35 years and above	2 (4.2)	0 (0.0)		

However, presence or otherwise of severe pain during menstruation (*χ* ^2^ = 0.471, *p* = 0.493), type of menstrual cycle (either regular or irregular) (*χ* ^2^ = 0.182, *p* = 0.670), age at 1^st^ Menstruation (*χ* ^2^ = 11.19, *p* = 0.0600) and age at 1st Delivery (*χ* ^2^ = 2.456, *p* = 0.402) were not significantly associated with confirmed uterine fibroids.

## Discussion

A total of 244 trans-abdominal pelvic ultrasound scan images of women were analysed. The age range, mean and standard deviation (SD) of patients were 14–54 years, 31.9 years and ± 7.9 respectively. Prior to the study, 35.2 % of the sampled participant’s stated that they had been previously diagnosed to have fibroids. However, during the study, only 71.1 % of that number were confirmed of having fibroids. Additionally, out of the 158 patients who did not know previously of having fibroid, 28.9 % were confirmed to have fibroid. In general the prevalence or confirmed rate of the participants having fibroid was 36.9 %. The finding is consistent with available data [14] which suggest that fibroids are the most common pelvic tumour in women, causing symptoms in approximately 25 % of reproductive age women with the overall prevalence of fibroids increasing to over 70 %.

From the study, 23 % (38/168) of women <35 had prevalent fibroids, compared to 67 % (36/54) of women 35–44, and 73 % (16/22) of women at 45 or above years. This study indicated that the highest prevalence of the fibroid cases was found among older women in the reproductive age group. The trend of fibroid prevalence in this study is consistent with findings of Lurie et al.*,* [16] which estimated the prevalence of uterine fibroid as 4 % in women aged 20–30 years, 11 to 18 % in women between 30–40 years and 33 % in women between 40–60 years. Having fibroids may result in menorrhagia, pelvic pain, urinary obstructive symptoms, infertility, and pregnancy loss [20]. The recorded situation in Ghana is worrying in view of health and other risk implications. Fibroid has social, economic and medical implications in the female populace. Premenopausal women (18–45 years) in Ghana constitute about 40 % of the Ghanaian population [17] and are strong component of the country’s workforce and thus contribute immensely to the economy.

Close to half 112 (45.9 %) of the patients were Akans ethnic groups. This finding is not surprising because according to the Ghana Statistical Service [31] Akans constitutes 47.5 % of the Ghanaian population. Despite the Akan ethnic group being the majority in this work, the study also showed that the Ga ethnic grouping (54.5 %) had the highest percentage of women with fibroids. A study by Biritwum, Gyapong and Mensah [32] indicated that the prevalence of obesity is highest among the Ga tribe and that could have accounted for the high incidence of fibroid among them since obesity is found to be a high risk factor for developing fibroids.

This study showed significant associations between age group of patients and the presence of uterine fibroids. The prevalence increased with increasing age and agrees with several epidemiologic studies [24, 33]. Thus, it may be implied that the age of an individual in the reproductive age plays a vital role in development of fibroids.

Factors that also proved to be associated with fibroid were age (years) at last delivery and BMI of participants. The above findings as reported in Table 3 suggest that uterine fibroid occurrences was common in obese patients and common in patients with late age at last delivery.

For BMI, several studies have found an association between obesity and an increased incidence of uterine fibroids [7]. This apparent association between obesity and fibroids may be related to hormonal factors associated with obesity, but other pathologic pathways might also be involved. Several relevant hormonal associations with obesity are known. A significant increase occurs in the conversion of circulating adrenal androgens to oestrogen by excess adipose tissue. The hepatic production of sex hormone-binding globulin is decreased, resulting in more unbound physiologically active oestrogen. Because almost all circulating oestrogen postmenopausally are derived from metabolism of circulating androgens by peripheral tissues, including fat, these two mechanism probably have more impact in premenopausal women [34]. In obese premenopausal women, decreased metabolism of estradiol by the 2-hydroxylation route reduces the conversion of estradiol to inactive metabolites, which could result in a relatively hyper oestrogenic state [35].

The negative association between parity and the risk of fibroids in this study is consistent with the findings in previous studies [26, 36, 37]. An explanation for this finding is that pregnancy reduces the time of exposure to unopposed oestrogens, whereas nulliparity or reduced fertility may be associated with an ovulatory cycle characterized by long term unopposed oestrogens. The alternate possibility exist that uterine fibroids are actually the cause of infertility, rather than the consequence of it; however, the diminished association of fibroids with parity remains essentially the same after exclusion of women with a history of infertility [14].

Meanwhile, factors such as presence or otherwise of severe pain during menstruation and type of menstrual cycle of participants (either regular or irregular), age at 1st Menstruation and age at 1st Delivery did not show any statistical significance. These findings suggest that the aforementioned factors are not necessarily factors that are commonly associated with uterine fibriod among Ghanaian women in this study.

## Conclusion

The study concludes that uterine fibroid in Ghanaian women is mainly associated with women of older age group of the reproductive age than the younger age categories and also associates more with women with no children as compared to those with more children. Moreover, it associates more with obese patients and patient with late age at last delivery. The Ghanaian woman is engaged in governmental and non-governmental businesses, which has seen a tremendous growth which is an indication that women are gradually taking up more equally demanding responsibilities aside their traditional roles. Therefore, managing the health of the Ghanaian woman is essential for the socio-economic growth of the country. The findings may aid in appropriate interventions and policy planning in the country.

## Abbreviations

BMI: body mass index
MHz: megahertz
OR: odd ratio
SD: standard deviation

## References

[1] Goodwin SC, Spices JB, Worthington-Kirsch R, Peterson E, Prong LS, Myers ER (2008). Uterine Artery Embplization For Treatment of leiomyomata: Long Term Out comes From Fibroid Registry. Obset Gynecol.

[2] Fledderjohann JJ (2012). “Zero is not good for me”: implications of infertility in Ghana. Hum Reprod.

[3] Levy B, Mukhejee T, Hirschhorn K (2000). Molecular cytogenetic analysis of uterine leiomyomas and leimyosarcoma by comparative genomic hybridization. Cancer Cytogenet.

[4] Viswanathan M, Hartmann K, McKoy N, et al. Management of Uterine Fibroids: An update of evidence. Agency for Healthcare Research and Quality (AHRQ): Summary, Evidence Report/Technology Assessment 2007; Number 154. AHRQ Publication No. 01-E051.

[5] Jennelle CH, Bradley JQ, Mark AR, Elizabeth AS, Paola Dal C, Cynthia CM (2008). Molecular and Cytogenetic Characterization of PlexiformLeiomyomata Provide Further Evidence for Genetic Heterogeneity Underlying Uterine Fibroids. Am J Pathol.

[6] Northington GM, Arya LA (2006). Uterine Leiomyoma. Obstetrics and Gynecology. Board Review Manual. Obstet Gynecol.

[7] Flake GP, Andersen J, Dixon D (2003). Etiology and Pathogenesis of Uterine Leiomyomas: A Review. Environ Health Perspect.

[8] Fiore K (2011). Fibroid Surgery May Up Birth Rates After Recurrent Miscarriage.

[9] Wilcox LS, Koonin LM, Pokras R, Strauss LT, Xia Z, Peterson HB (1994). Hysterectomy in the United States, 1988–1990. Obstet Gynecol J.

[10] Velebil P, Wingo PA, Xia Z (1995). Rate of hospitalization for gynecologic disorders among reproductive-age women in the United States. Obstet Gynecol.

[11] Chao-Ru C, Germaine MB, Norman GC, Kimberly MP, Jean W-W (2001). Risk Factors for Uterine Fibroids among Women Undergoing Tubal Sterilization. Am J Epidemiol.

[12] Neiger R, Sonek J, Croom C, Ventolini G (2006). Pregnancy-related changes in the size of uterine leiomyomas. J Reprod Med.

[13] Haney AF (2000). Clinical decision making regarding leiomyomata: what we need in the next millennium. Environ Health Perspect.

[14] Goodwin SC (2009). Spies JB Uterine fibroid embolization. N Engl J Med.

[15] Jacobson GF, Shaber RE, Hung YY (2007). Changes in rates of hysterectomy and uterine conserving procedures for treatment of uterine leiomyoma. Am J of Obstet Gynecol.

[16] Lurie S, Piper I, Woliovitch I, GleZeman M (2005). Age Related Prevalence of Sonographically Confirmed Uterine Myoma. J Obstet Gynaecol.

[17] Masters C Are hysterectomies too common? TIME Magazine [http://www.time.com/time/health/article/0,8599,1644050,00.html?cnn=yes], Accessed October 27, 2009.

[18] Xu L, Wang Y, Collins CD, Tang S (2007). Urban health insurance reform and coverage in China using data from National Health Services Surveys in 1998 and 2003. BMC Health Serv Res.

[19] Van Voorhis BJ, Romitti PA, Jones MP (2002). Family history as a risk factor for development of uterine leiomyomas: results of a pilot study. J Reprod Med.

[20] Han PK (1997). Historical changes in the objectives of the periodic health examination. Ann Intern Med.

[21] Broder MS, Kanouse DE, Mittman BS, Bernstein SJ (2000). The appropriateness of recommendations for hysterectomy. Obstet Gynecol.

[22] Boulware LE, Marinopoulos S, Phillips KA, Hwang CW, Maynor K, Merenstein D, Wilson RF, Barnes GJ, Bass EB, Powe NR, Daumit GL (2007). Systematic review: the value of the periodic health evaluation. Ann Intern Med.

[23] Marshall LM, Spiegelman D, Manson JE (1998). Risk of uterine leiomyomata among premenopausal women in relation to body size and cigarette smoking. Epidemiology.

[24] Wu Hai-Yun, Ling-Ling Yang, Shan Zhou Impact of periodic health examination on surgical treatment for uterine fibroids in Beijing: a case control study. BMC Health Services Research 2010, (Assessed on 20th Dec, 2012 at http://www.biomedcentral.com/1472-6963/10/329). 10.1186/1472-6963-10-329 PMC3002351 21134290

[25] Faerstein E, Szklo M, Rosenshein NB (2001). Risk factors for uterine leiomyoma: a practice based case–control study. II. Atherogenic risk factors and potential sources of uterine irritation. Am J Epidemiol.

[26] Parazzini F, Negri E, La Vecchia C, Chatenoud L, Ricci E, Guarnerio P (1996). Reproductive factors and risk of uterine fibroids. Epidemiology.

[27] Segars JH (2008). Uterine Fibroid. National Inst Child Health Hum Dev.

[28] Boynton-Jarrett R, Rich-Edwards J, Malspeis S, Missmer SA, Wright R (2005). A prospective study of hypertension and risk of uterine leiomyomata. Am J Epidemiol.

[29] Wise LA, Palmer JR, Reich D, Cozier YC, Rosenberg L (2012). Hair Relaxer Use and Risk of Uterine Leiomyomata in African-American Women. Am J Epidemiol.

[30] Baird DD, Dunson DB, Hill MC, Cousins D, Schectman JM (2007). Association of physical activity with development of uterine leiomyoma. Am J Epidemiol.

[31] Ghana Statistical Service, Ghana Statistical Services (GSS), Noguchi Memorial Institute for Medical Research (NMIMR), and ORC Macro. Ghana demographic and health survey. 2003.

[32] Biritwum RB, Gyapong J, Mensah G (2005). The Epidemiology of Obesity in Ghana. Ghana Med J.

[33] Sarkodie BD, Botwe OB, Ofori EK. Uterine fibroid characteristics and sonographic pattern among Ghanaian females undergoing pelvic ultrasound scan: a study at 3-major centres. BMC Women's Health. 2016;16:10. doi:10.1186/s12905-016-0288-4. 10.1186/s12905-016-0288-4 PMC4754812 26884233

[34] Glass AR (1989). Endocrine aspects of obesity. Med Clin North Am.

[35] Schneider J, Bradlow HL, Strain G, Levin J, Anderson K, Fishman J (1983). Effects of obesity on estradiol metabolism: decreased formation of nonuterotropic metabolites. J Clin Endocrinol Metab.

[36] Cramer SF, Horiszny JA, Leppert P (1995). Epidemiology of uterine leiomyomas with an etiologic hypothesis. J Reprod Med.

[37] Lumbiganon P, Rugpao S, Phandhu-Fung S, Laopaiboon M, Vudhikamraka N, Werawatakul Y (1996). Protective effect of depot-medroxyprogestrone acetate on surgically treated uterine leiomyomaa: a multicentre case–control study. Br J Obstet Gynaecol.

